# Clinicopathologic Characteristics of Oestrogen Receptor-Positive/Progesterone Receptor-Negative/Her2-Negative Breast Cancer According to a Novel Definition of Negative Progesterone Receptor Status: A Large Population-Based Study from China

**DOI:** 10.1371/journal.pone.0125067

**Published:** 2015-05-04

**Authors:** An-qi Li, Shu-ling Zhou, Ming Li, Yan Xu, Ruo-hong Shui, Bao-hua Yu, Wen-tao Yang

**Affiliations:** 1 Department of Pathology, Fudan University Shanghai Cancer Center, Shanghai, P.R. China; 2 Department of Oncology, Shanghai Medical College, Fudan University, Shanghai, P.R. China; University of South Alabama, UNITED STATES

## Abstract

**Purpose:**

A lack of progesterone receptor (PgR) expression in oestrogen receptor-positive (ER+) tumours is associated with worse survival. PgR status is usually defined as positive or negative using 1% positive nuclei as a cut-off point. In this study, we aimed to assess the clinicopathologic characteristics of ER+/PgR-/HER2- tumours by comparing them with ER+/PgR+/HER2- tumours using a PgR cut-off point of 20% as a divisive criterion.

**Methods:**

We analysed 1,522 patients with primary breast cancer who had undergone surgery at the Cancer Center of Fudan University between 2012 and 2014. Age, grade, tumour size, lymph node status and lymphovascular invasion were assessed. Multinomial logistic regression, linear regression and chi-square test models were applied to assess associations between ER, PR and clinical features.

**Results:**

ER+/PgR-/HER2- tumours showed poorer clinicopathologic characteristics relative to ER+/PgR+/HER2- tumours using a PgR threshold of 20% instead of 1%. The clinicopathologic characteristics did not differ between tumours with purely negative PgR expression and tumours with a PgR percentage ranging from 1% to 19%. The prognostic significance of PR expression appeared more pronounced in patients under a high Ki-67 status than those under a low Ki-67 status.

**Conclusions:**

Based on these findings, we propose the use of a novel threshold of 20% to define PgR status. Nevertheless, the impact of this new criterion on patient management and clinical treatment requires additional study.

## Introduction

Oestrogen and oestrogen receptors (ERs) play key roles in both normal breast development and breast cancer progression. ER expression is a prognostic factor and powerful indicator of endocrine responsiveness in the clinical management of breast cancer. Previous reports have shown that substantial progesterone receptor (PgR) positivity in tumours is commonly associated with a better prognosis [1,2]. However, the ability of PgR expression to predict a benefit for endocrine therapy remains controversial. In 1975, researchers first hypothesised that PgR could predict the response to endocrine therapy [3]. Later, the PgR status was verified to significantly improve outcome prediction over ER status alone for adjuvant endocrine therapy [4]. However, a few studies have suggested that the recurrence and death rate ratio is independent of PgR status in ER-positive (ER+) disease treated with adjuvant tamoxifen and that luminal A and B tumours similarly benefit from endocrine therapy regardless of PgR expression [5,6]. The absence of PgR expression indicates a higher risk of relapse [7] and is associated with poor survival outcome [8]. A previous study showed that ER+/PgR-negative (PgR-) tumours displayed more aggressive characteristics than ER+/PgR-positive (PgR+) tumours [9]. Furthermore, ER+/PgR- tumours expressed higher levels of HER1 and HER2 than ER+/PgR+ tumours [9]. However, many studies of ER+/PgR- cases did not exclude the positive expression of HER2, which may strongly impact the clinical characteristics and prognosis [10].

Depending on the gene and protein expression differences between luminal A and B tumours and their clinicopathologic features and survival outcomes, Prat et al. [2] proposed an empiric cut-off point of 20% for PgR to better distinguish luminal A from luminal B breast cancer. This definition was adopted by the panel at the 2013 St Gallen International Breast Cancer Conference [11]. Later, Maisonneuve et al. [12] verified the accuracy of this new surrogate definition of luminal subtypes in terms of distant disease control, which supports the newly proposed threshold of 20% of PgR.

In this study, we adopted the PgR threshold of 20% as a criterion to categorise patients with “low” (<20%) PgR expression into an ER+/PgR-/HER2- group. We also validated the accuracy of this classification and aimed to further elucidate the clinicopathologic features of ER+/PgR-/HER2- tumours by comparing them with ER+/PgR+/HER2- tumours.

## Materials and Methods

### Ethics statement

This was a retrospective study. All the specimens were retrieved from the Pathology Department of the Cancer Center, Fudan University. The study was approved by the independent ethics committee/institutional review board of Fudan University Shanghai Cancer Center (Shanghai Cancer Center Ethical Committee). Informed consent was waived by the ethics committee.

### Study population

This study was based on a cohort of 1,522 patients who had undergone surgery and were diagnosed with primary invasive breast carcinoma of no special type (NST) at the Pathology Department of the Cancer Center, Fudan University, Shanghai, China, between 2012 and 2014. Patients who had received neoadjuvant therapy were excluded. HER2-positive cases were excluded. The clinicopathologic features, including patient age, histologic grade, tumour size, lymph node status (including sentinel lymph node, LN), lymphovascular invasion (LVI), and expression of ER, PgR, HER2 and Ki67 were extracted from the original reports.

Immunostains for ER, PgR, HER2, Ki-67 were performed on formalin-fixed, paraffin-embedded tissues. The primary antibodies used in this study were obtained from commercial sources (ER, PgR, HER2, and Ki-67 from Roche, Swiss). The ER, PgR, HER2 and Ki-67 assays were performed on the BenchMark XT autostainer (Ventana) based on the avidin-biotin complex method.

The expression levels of ER, PgR and Ki-67 were scored based on the percentage of nuclear staining in invasive tumour cells. ER was considered positive when ≥1% nuclei stained, as proposed by the 2010 American Society of Clinical Oncology/College of American Pathologists (ASCO/CAP) guidelines [13]. Samples were considered positive for PgR or Ki-67 in cases that scored ≥20%. The expression of HER2 was evaluated on a standardised scale from 0–3 based on the intensity of membranous staining and the proportion of staining of invasive tumour cells, and strong complete membranous staining in >30% of tumour cells (3+) was considered positive according to the 2007 ASCO/CAP guidelines [14]. HER2 2+ tumours were further assessed using a fluorescent in situ hybridisation (FISH) detection of HER2/Neu gene amplification with the FDA-approved PathVysion HER2/Neu DNA Probe Kit (Abbott Laboratories). At least 20 invasive tumour cells in each slide were evaluated to determine the number of HER2 gene copies and the ratio of the HER2 gene to the chromosome 17 centromere signals. According to the 2007 ASCO/CAP recommendations [14], a HER2/CEP17 ratio >2.2 constitutes HER2 gene amplification.

### Statistical analysis

Statistical analyses was performed using SPSS 20.0 statistical software (SPSS Inc, Chicago, IL). Significant differences in the clinicopathologic features between groups were evaluated using the chi-squared test. Multivariate analyses of PgR relative to various factors were performed with a multinomial logistic regression model, which yielded the HR and 95% CI for each variable. Linear regression and chi-square test models were applied to investigate the shape of the relationship among patient age, tumour size, LN status, LVI, histologic grade, expression of Ki67 and quantitative ER/PgR expression. The age and expression of ER/PgR were treated as continuous variables for association estimates. All statistical tests were two-sided, and *P* values of 0.05 or less were considered significant.

## Results

**Fig 1:**
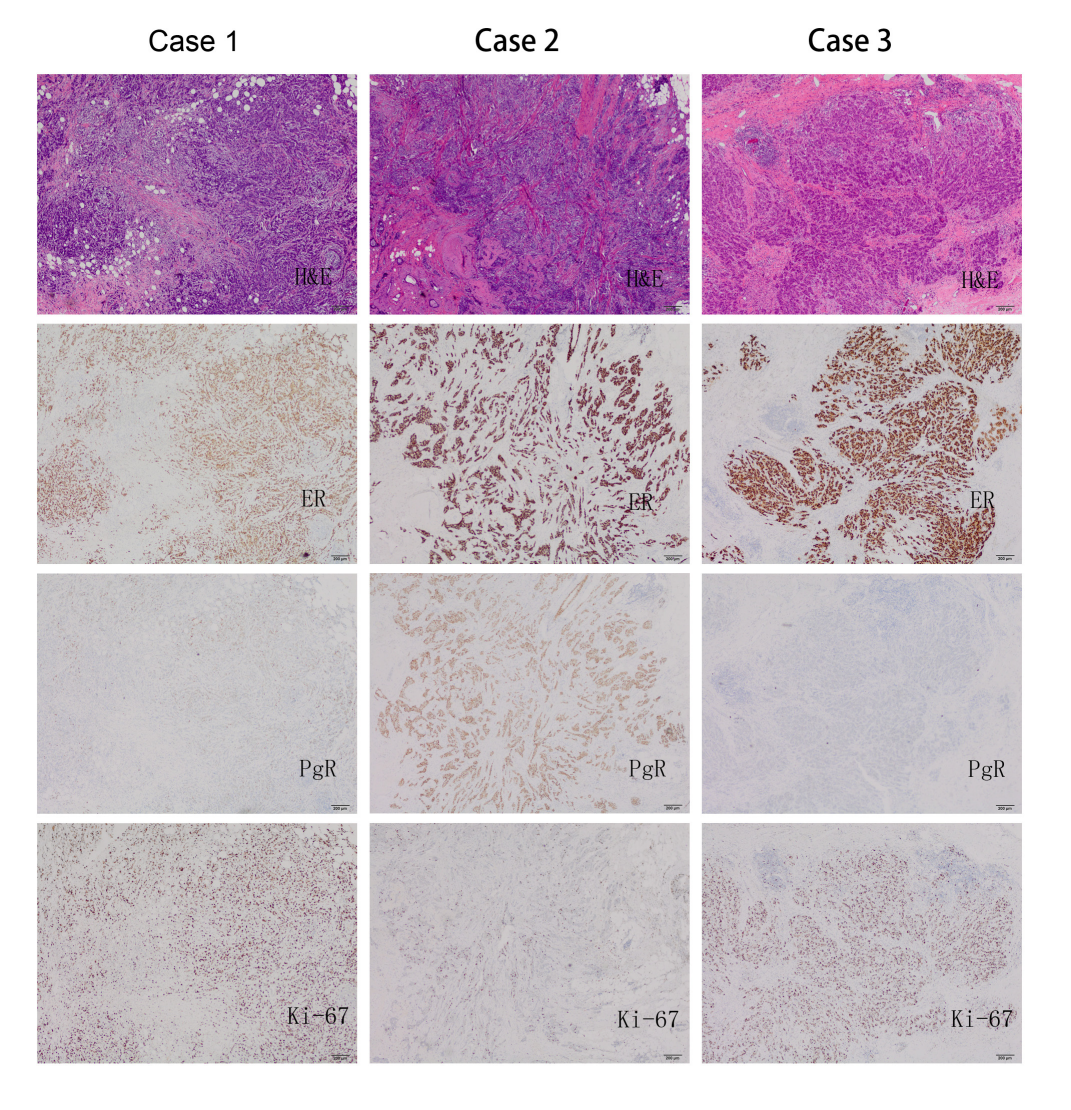
Representative findings of H&E staining and IHC for ER, PgR, Ki-67. In case 1, a patient with Grade II tumour scored 95%, 15%, and 80% for ER, PgR, and Ki-67, respectively. In case 2, a patient with Grade II tumour scored 95%, 95%, and 10% for ER, PgR, and Ki-67, respectively. In case 3, a patient with Grade III tumour scored 90%, 0%, and 70% for ER, PgR, and Ki-67, respectively (magnification x400).

In the current study, 1156 cases (76.0%) were ER+/PgR+/HER2-, and 366 (24.0%) cases exhibited an ER+/PgR-/HER2- phenotype. The subtypes were designated based on the results of ER, PgR, Ki-67 and HER2 staining according to the 2013 St Gallen International Breast Cancer Conference. A series of 479 (31.5%) patients with ER+, PgR+, HER2- and Ki-67 staining <20% was categorised as luminal A subtype, and 1043 (68.5%) patients with ER+ and at least with ‘high’ Ki-67 or ‘negative or low’ PgR expression were categorised as luminal B HER2 negative. Among the patients with PgR- tumours, 154 tumours were purely negative for PgR, and the PgR percentage of 212 tumours ranged from 1% to 19%. Representative findings of haematoxylin-eosin staining (H&E) and IHC for ER, PgR, Ki-67 are shown in Fig 1.

### Comparison of ER+/PgR+/HER2- and ER+/PgR-/HER2-group in terms of clinicopathologic characteristics

The clinicopathologic variables included the patient gender, age, histologic grade, tumour size, LN status, status of LVI and expression of Ki-67 (Table 1). Compared with ER+/PgR-/HER2-, the ER+/PgR+/HER2- group generally exhibited more favourable clinicopathologic characteristics. Most patients in the ER+/PgR–/HER2- group were older (median age, 55.1 years) than those in the ER+/PgR+/HER2- group (median age, 51.3 years; *P* = 0.009). Grade III tumours (*P* < 0.0001), larger tumours (≥5 cm, *P* < 0.0001) and tumours in pN3 (*P* = 0.002) were more common in the ER+/PgR-/HER2- group, whereas tumours in pN1 (*P* = 0.003) were more frequently observed in the ER+/PgR+/HER2- group. LVI did not differ between groups. Multivariate analysis demonstrated that PgR was independently associated with older age (hazard ratio [HR], 2.48; 95% confidence interval [CI], 1.931–3.185; *P* < 0.0001), grade III tumours (HR, 2.118; 95% CI, 1.581–2.839, *P* < 0.0001), larger tumours (≥5 cm, HR, 4.831; 95% CI, 1.664–14.024, *P* = 0.004), tumours in pN3 (HR, 2.002; 95% CI, 1.021–3.922, *P* = 0.043) and high Ki-67 (HR, 1.391; 95% CI, 1.046–1.850; *P* = 0.023).

**Table 1:** Comparison between groups in terms of clinicopathologic characteristics.

Variable	Total		ER+/PgR+/HER2-		ER+/PgR-/HER2-		
	No.	%	No.	%	No.	%	*P*
**Gender**							
Male	5	0.3%	1	0.1%	4	1.1%	0.003
Female	1517	99.7%	1155	99.9%	362	98.9%	
**Age, years**							
**Range**	22–93		22–93		24–85		
<60	1139	74.8%	884	76.5%	255	69.7%	0.009
≥60	383	25.2%	272	23.5%	111	30.3%	
**Histologic grade**							
I/ II	1160	76.2%	921	79.7%	239	65.3%	<0.0001
III	362	23.8%	235	20.3%	127	34.7%	
**Tumour size, cm**							
T<2	584	38.4%	446	38.6%	138	37.7%	
2≤T<5	922	60.6%	704	60.9%	218	59.6%	0.001
T≥5	16	1.1%	6	0.5%	10	2.7%	
**LN status**							
pN0 (none)	912	59.9%	685	60.1%	227	64.1%	
pN1 (1–3 LN)	391	25.7%	320	28.1%	71	20.1%	
pN2 (4–9 LN)	128	8.4%	97	8.5%	31	8.8%	0.001
pN3 (≥10 LN)	62	4.1%	37	3.2%	25	7.1%	
pNX^a^	29	1.9%	17	-	12	-	
**LVI**							
Negative	945	62.1%	717	62.0%	228	62.3%	0.926
Positive	577	37.9%	439	38.0%	138	37.7%	
**Ki-67, %**							
1–19	591	38.8%	479	41.4%	112	30.6%	<0.0001
≥20	931	61.2%	677	58.6%	254	69.4%	

^a^Patients with unknown LN status underwent lumpectomy or conserving surgery.

### Relevance of measured ER and PgR status to clinicopathologic characteristics

**Fig 2:**
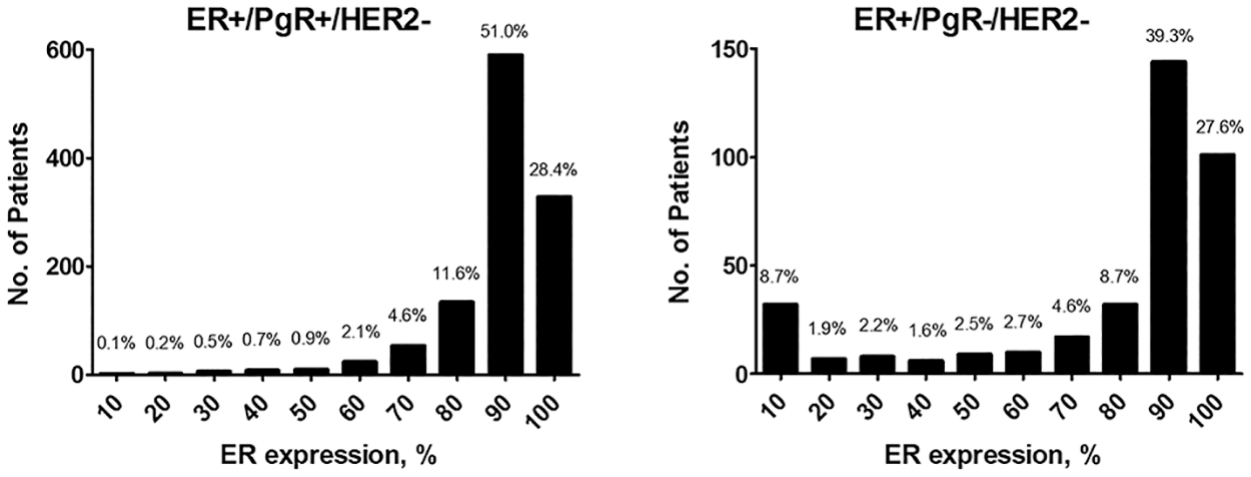
Distribution of ER expression as percentage of immunoreactive cells for the PgR-positive and PgR-negative groups. Histogram bars are in 10-unit bins, beginning with 1% of cells, 1% to 10%, 11% to 20%, etc. The ER+/PgR+/HER2- group consisted of 27 (2.3%) patients with ER <50% and 1129 (97.7%) patients with ER ≥50%. The ER+/PgR-/HER2- group consisted of 62 (16.9%) patients with ER <50% and 304 (83.1%) patients with ER ≥50%. The number of patients with a higher level of ER expression (ER ≥50%) in the ER+/PgR+/HER2- group was significantly larger that in the ER+/PgR-/HER2- group (*P* < 0.0001).

**Fig 3:**
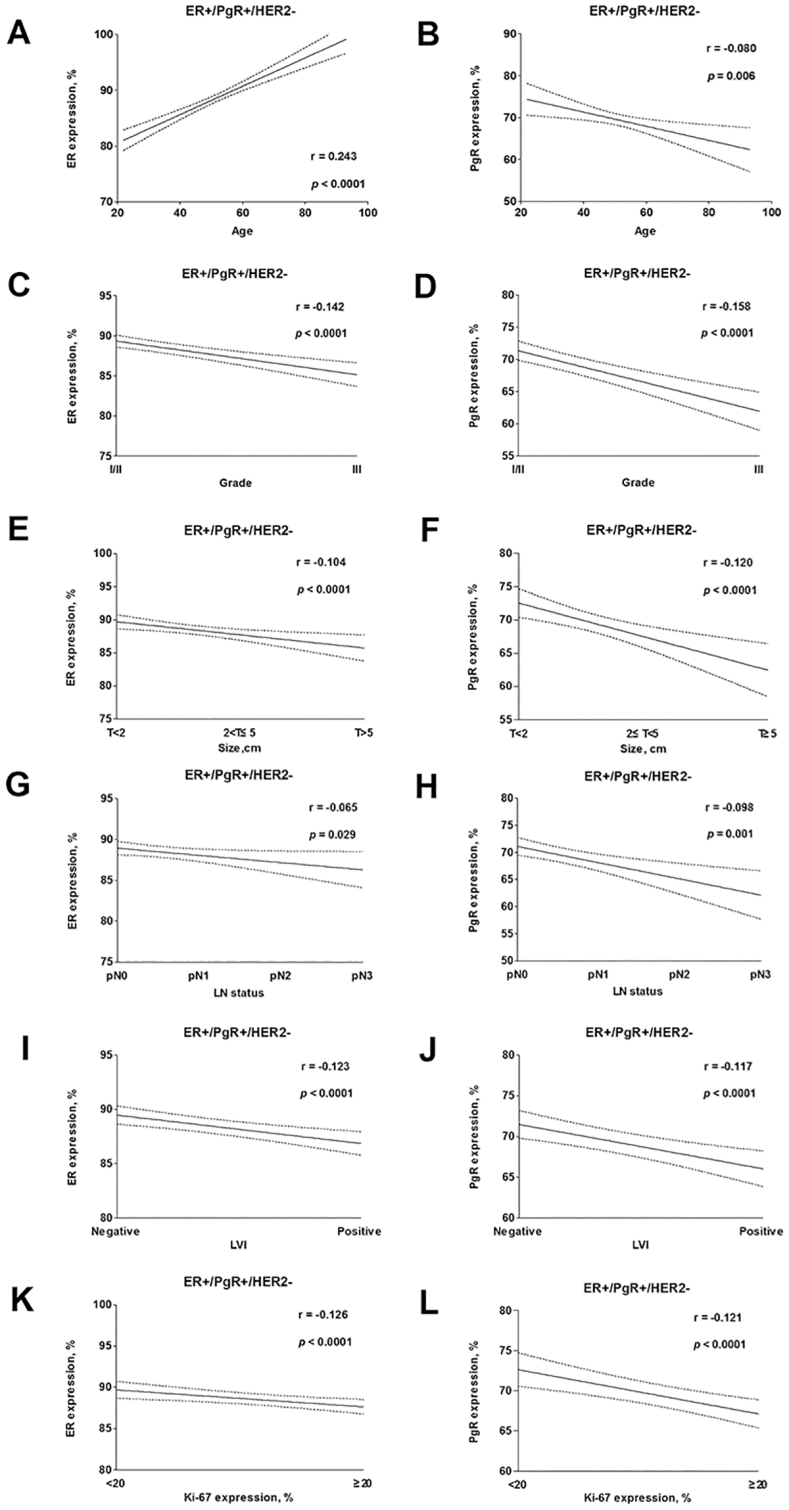
Good overall correlation was observed between ER and PgR expression with clinicopathologic variables among ER+/PgR+/HER2- (a-l) tumours. ER expression levels were positively correlated with age, whereas PgR expression levels were negatively correlated with age. ER and PgR expression levels were both negatively correlated with grade, size, LN status, Ki-67 and LVI.

In the ER+/PgR+/HER2- group, the ER percentage ranged from 10% to 100% (median, 88.5%), and the PgR percentage ranged from 20% to 100% (median, 69.1%); none of the tumours expressed ER in the range of 1% to 9%. In the ER+/PgR-/HER2- group, the ER percentage ranged from 1% to 100% (median, 71.8%), and the tumours of 22 patients expressed ER at levels <10%, whereas the PgR percentage ranged from 0% to 15% (median, 3.3%). The distributions of ER scores in two groups are displayed in Fig 2. Higher ER expression (ER ≥ 50%) was more common in the ER+/PgR+/HER2- group than the ER+/PgR-/HER2- group (P < 0.0001). An association between ER, PR expression and clinicopathologic variables was observed in ER+/PgR+/HER2- tumours (Fig 3). The expression levels of ER and PR were directly correlated with the favourability of the clinicopathologic characteristics in ER+/PgR+/HER2- tumours.

### Predictive role of PgR under high or low Ki-67 status

The differences between luminal A and luminal B tumours depend on the choice of the threshold value for Ki-67 and the requirement for PgR positivity. Therefore, we aimed to determine optimal threshold values and whether a lack of substantial PgR positivity differs from being PgR-positive as a function of the Ki-67 status (high or low). The results are displayed in Table 2. None of the variables significantly differed between the PgR+ and PgR- group when Ki-67 expression was low. Interestingly, the opposite results were found for the high Ki-67 status, except for LVI status.

**Table 2:** Clinicopathologic characteristics in patients according to PgR and Ki-67 status.

Variable	ER+/HER2-/Ki-67<20%					ER+/HER2-/Ki-67≥20%				
	PgR+		PgR-		*P*	PgR+		PgR-		*P*
	No.	%	No.	%		No.	%	No.	%	
**Total**	479		112			677		254		
**Age, years**										
**Range**	25–93		27–85			22–82		24–83		
**Mean age**	52.5		56.5			50.4		54.5		
<60	349	72.9%	72	64.3%	0.071	535	79.0%	183	72.0%	0.024
≥60	130	27.1%	40	35.7%		142	21.0%	71	28.0%	
**Histologic grade**										
I/ II	447	93.3%	108	96.4%	0.216	474	70.0%	131	51.6%	<0.0001
III	32	6.7%	4	3.6%		203	30.0%	123	48.4%	
**Tumour size, cm**										
T<2	212	44.3%	58	51.8%		234	34.6%	80	31.5%	
2≤T<5	264	55.1%	53	47.3%	0.325	440	65.0%	165	65.0%	0.001
T≥5	3	0.6%	1	0.9%		3	0.4%	9	3.5%	
**LN status**										
pN0 (none)	316	67.7%	79	75.2%		369	54.9%	148	59.4%	
pN1 (1–3 LN)	119	25.5%	19	18.1%		201	29.9%	52	20.9%	
pN2 (4–9 LN)	25	5.4%	5	4.8%	0.424	72	10.7%	26	10.4%	0.009
pN3 (≥10 LN)	7	1.5%	2	1.9%		30	4.5%	23	9.2%	
pNX^a^	12	-	7	-		5	-	5	-	
**LVI**										
Negative	344	71.8%	85	75.9%	0.384	373	55.1%	143	56.3%	0.742
Positive	135	28.2%	27	24.1%		304	44.9%	111	43.7%	

^a^Patients with unknown LN status underwent lumpectomy or conserving surgery.

### Comparison between the purely negative and low PgR expression groups

To verify the accuracy of the proposed PgR threshold, several variables, including histologic grade, tumour size, LN status, and status of LVI, were evaluated in the purely negative and low (1 ≤ PgR < 20%) PgR groups (Table 3). None of the examined variables significantly differed between groups.

**Table 3:** Comparison between the purely negative and low PgR expression groups.

Variable	ER+/PgR-/HER2-		ER+/PgR<20%/HER2-		
	No.	%	No.	%	*P*
**Total**	154	42.08%	212	57.92%	
**Age, years**					
**Range**	24–83		27–85		
**Mean age**	54.9		55.2		
<60	105	68.18%	150	70.75%	0.591
≥60	49	31.82%	62	29.25%	
**Histologic grade**					
I/ II	92	59.74%	147	69.34%	0.057
III	62	40.26%	65	30.66%	
**Tumour size, cm**					
T<2	64	41.56%	74	34.91%	
2≤T<5	87	56.49%	131	61.79%	0.356
T≥5	3	1.95%	7	3.30%	
**LN status**					
pN0 (none)	98	66.67%	129	62.32%	
pN1 (1–3 LN)	25	17.01%	46	22.22%	
pN2 (4–9 LN)	12	8.20%	19	9.20%	0.582
pN3 (≥10 LN)	12	8.20%	13	6.30%	
pNX^a^	7	-	5	-	
**LVI**					
Negative	102	66.23%	126	34.91%	0.185
Positive	52	33.77%	86	61.79%	
**Ki-67, %**					
1–19	47	30.52%	65	30.66%	0.977
≥20	107	69.48%	147	69.34%	

^a^Patients with unknown LN status underwent lumpectomy or conserving surgery.

## Discussion

At the 2013 St Gallen International Breast Cancer Conference, the cut-off point for PgR was increased from 1% to 20% to improve the definition of luminal A breast cancer [11]. Consequently, the number of patients classified as luminal A decreased, and the number of patients for whom cytotoxic therapy is generally recommended increased. To the best of our knowledge, a comprehensive evaluation of the clinicopathologic characteristics of ER+/PgR–/HER2- breast cancer in relation to ER+/PgR+/HER2- tumours has not been published since the release of the 2013 St Gallen guidelines. Thus, we attempted to evaluate the prognostic role of PgR using a cut-off point of 20% and determine whether this threshold is appropriate to differentiate PgR-positive from PgR-negative disease and correctly elucidate the clinicopathologic features of ER+/PgR+/HER2- and ER+/PgR-/HER2- tumours.

Our results primarily confirm that patients with ER+/PgR-/HER2- tumours display more unfavourable clinicopathologic characteristics compared with patients with ER+/PgR+/HER2- tumours, which affirms the prognostic importance of PgR expression. ER+/PgR-/HER2- tumours were observed in older patients at diagnosis, and these tumours were larger, generated more metastatic lymph nodes, a lower level of ER expression and a higher proliferation rate. These features are concordant with previously published studies, although the PgR threshold in these studies was 1% [1,9]. However, some reports have shown that PgR expression is not correlated with the LN status or tumour size, which is inconsistent with our results [7,15]. These differences may be due to an unselected breast cancer population without the exclusions of patients who received neoadjuvant therapy or who expressed HER2 positivity.

In addition, we have investigated the relationship between the quantitative ER and PgR expression levels and the clinicopathologic characteristics in detail. ER+/PgR-/HER2- tumours presented with a lower level of ER expression than ER+/PgR+/HER2- tumours, independent of the clinical tumour characteristics. This finding indicates the aetiology of ER+/PgR- tumours, which is currently unclear. Many theories have previously been proposed to explain the biology of PgR loss in ER+ breast cancer [16]. PgR, an oestrogen-regulated gene, requires oestrogen and ER for its synthesis in normal and cancer cells. Therefore, the existence of ER-/PgR+ tumours remains controversial and ER-/PgR+ tumours are not classified into any of the subtypes according to the 2013 St Gallen guidelines [11]. Our study identified clear associations between ER, PgR and clinicopathologic characteristics. The presence of ER is generally agreed to be a favourable prognostic factor, and PgR expression was later shown to add significant prognostic value in breast cancer beyond that obtained with ER alone [17]. A previous study reported that the presence of LVI is associated with poor outcome and indicated that LVI is a powerful independent prognostic factor [18]. We found that the presence of LVI is associated with both ER and PR expression in our study; however, the expression level of LVI did not significantly differ between the ER+/PgR+/HER2- and ER+/PgR-/HER2- groups, and this finding agrees with that of a previous study [15].

When our cases were stratified according to Ki-67, no significant differences were observed between the ER+/PgR+/HER2- and ER+/PgR-/HER2- groups based on a threshold of Ki-67 <20%. However, significant differences were observed between two groups in terms of age, grade, size, and LN status when considering this threshold. These results attracted our attention. Patients with ER+/PgR-/HER2-/ Ki-67 <20% tumours were once classified as luminal A patients who may only require endocrine therapy. Now, these patients may not be able to avoid adjuvant chemotherapy, given their luminal B cancer status according to the 2013 St Gallen International Breast Cancer Conference [11]. In our ER+/PgR-/HER2-/ Ki-67 <20% group, patients with luminal B tumours showed no significant difference in terms of all the prognostic factors when comparing to patients with ER+/PgR+/HER2-/ Ki-67 <20% tumours. Generally, luminal A disease has better outcome and requires only endocrine therapy. Thus, we suppose that patients in the ER+/PgR-/HER2-/ Ki-67 <20% group would have similar prognosis. A recent study showed that the distant disease-free survival (DDFS) of patients with Ki-67 <14% and PgR <20% were similar to those of patients with Ki-67 <14% and PgR ≥20% in ER+/HER2- tumours, which supports our assumption [12]. However, there is no direct evidence provided to support our statement. Thus, further studies of patients with ER+/PgR-/HER2-/ Ki-67 <20% tumours are needed. Many studies have shown that Ki-67 is a useful prognostic marker in early breast cancer [19,20]. However, this approach features certain limitations, such as thresholds ranging from 5 to 20% [21–23], tumour heterogeneity and poor interobserver agreement [24]. The majority of the Panel from the 2013 St Gallen voted that a threshold of ≥20% is clearly indicative of high Ki-67 status, whereas a minority still questioned this value and the role of Ki-67 in breast cancer treatment decisions. Maisonneuve et al. [12] recommended the use of Ki-67 <14% and ≥20% levels to define luminal A and B tumours, respectively, and classified tumours with intermediate (14–19%) Ki-67 levels to a further extent according to low PgR (< 20%) or high PgR (≥ 20%) status. However, we doubt the practicability of this definition because discriminating 14% from 20% is difficult.

Finally, we attempted to verify the accuracy of using the 20% threshold to define PgR positivity. None of the variables markedly differed between patients with ER+/PgR purely negative/HER2- tumours and ER+/PgR<20%/HER2- tumours. Recent studies have shown that patients with borderline to high ER with low PgR expression benefited more from chemotherapy plus tamoxifen compared with tamoxifen alone [25,26]. These findings support our suggestion that patients with low PgR should be classified into a PgR-negative group and treated with more aggressive adjuvant therapy. However, this finding also prompts another question: should patients from the ER+/PgR-/HER2-/ Ki-67 <20% group be treated with chemotherapy? Thus, this subtype must be studied further.

A potential limitation of our study should be mentioned. Our study population is consisted of patients selected since 2012; therefore, the follow-up is too short to evaluate the outcomes in each group. Thus, our team will further collect the patient outcomes and estimate the validity of our prognostic characteristics.

In summary, our study found that ER+/PgR-/HER2- tumours present more unfavourable clinicopathologic characteristics than ER+/PgR+/HER2- tumours. Assessing PR status using a threshold of 20% positive cells may improve our understanding of the clinicopathologic characteristics of ER+/PgR-/HER2- tumours in routine clinical practice and determine more appropriate treatments for patients with this tumour type.
